# Intercontinental migration pattern and genetic differentiation of arctic‐alpine *Rhodiola rosea* L.: A chloroplast DNA survey

**DOI:** 10.1002/ece3.4589

**Published:** 2018-11-23

**Authors:** Zsuzsanna György, Endre G. Tóth, Norbert Incze, Bence Molnár, Mária Höhn

**Affiliations:** ^1^ Department of Genetics and Plant Breeding Szent István University Budapest Hungary; ^2^ Institute of Forest Research University of Quebec at Abitibi‐Temiscamingue (IRF‐UQAT) Rouyn‐Noranda Quebec Canada; ^3^ Department of Botany Szent István University Budapest Hungary; ^4^ Applied Genomics Department, Agricultural Institute, Centre for Agricultural Research Hungarian Academy of Sciences Martonvásár Hungary

**Keywords:** arctic‐alpine species, chloroplast, genetic diversity, indels, noncoding regions, roseroot

## Abstract

Our study describes genetic lineages and historical biogeography of *Rhodiola rosea* a widely distributed arctic‐alpine perennial species of the Northern Hemisphere based on sequence analysis of six chloroplast regions. Specimens of 44 localities from the Northern Hemisphere have been sequenced and compared with those available in the GenBank. Our results support the migration of the species into Europe via the Central Asian highland corridor, reaching the European Alpine System (EAS) and also the western European edge, the British Isles. The EAS proved to be an important center of genetic diversity, especially the region of the Eastern Alps and the Dolomites where signs of glacial refugia was observed. Apart from those of the EAS, a common lineage was detected along the Atlantic coast from the British Isles toward Scandinavia as well as Iceland and the eastern parts of North America. Accordingly, the British Isles represent a main link between the northern Atlantic and southern EAS lineages.

## 1 INTRODUCTION

Phylogeographic studies performed on the European high mountain flora provided evidences for the Asian origin of many species that evolved and diversified on the highlands of the Qinghai‐Tibetian Plateau (QTP) and adjacent areas from where they have colonized the Northern Hemisphere (NH). Species’ migration route from the QTP toward Europe reaching the European Alpine System (EAS, the biogeographic region covering the Pyrenees, the Alps, the Carpathians and the Northern Balkans) and the nordic subarctic, arctic areas was proposed to follow the so‐called Central Asiatic highland corridor with the subsequently intervening high mountain ranges between Asia and Europe (Kadereit, Licht, & Uhink, 2008; Ohba, 1989). However, an expansion toward the northern Siberian region from the QTP was also mentioned for some species like *Saxifraga oppositifolia* (Abbott, Smith, & Milne, 2000) that by reaching Siberia and the Taymyr area—which might also served as a refugial territory—followed both an eastward and a westward migration route. Northern glacial survival and extensive postglacial migration in the Arctic region was also reported in *Vaccinium uliginosum* (Alsos, Engelskjøn, Gielly, Taberlet, & Brochmann, 2005). Moreover, some Scandivanian populations of *Dryas octopetala* provided evidence for the existence of a contact zone in the area where the colonizing European lineage most probably was intermingled with immigrants of the Northeast lineage (Alsos et al., 2007; Skrede, Eidesen, Pińeiro Portela, & Brochmann, 2006). The complex biogeographic connection of the QTP and the NH involves also intercontinental disjunction reaching North America, which was demonstrated in some herbaceous species inhabiting the arctic regions and high mountain elevations (Alsos et al., 2015). The radiation toward the new habitats, the evolution of genetic lineages and the different migration and colonization potential of species in response to the historical climatic oscillations and abiotic changes provided a complex biogeographic pattern in most taxa originating from the QTP (Zhang, Meng, Allen, Wen, & Rao, 2014). Moreover, studies on population level of single species across the entire range provided further insights into species’ phylogeography and evolution in relatively recent historical times involving the climate oscillations of the Quaternary (Christe et al., 2014; Schönswetter, Stehlik, Holderegger, & Tribsch, 2005; Taberlet, Fumagalli, & Wust‐Saucy, 1998).

**Figure 1:**
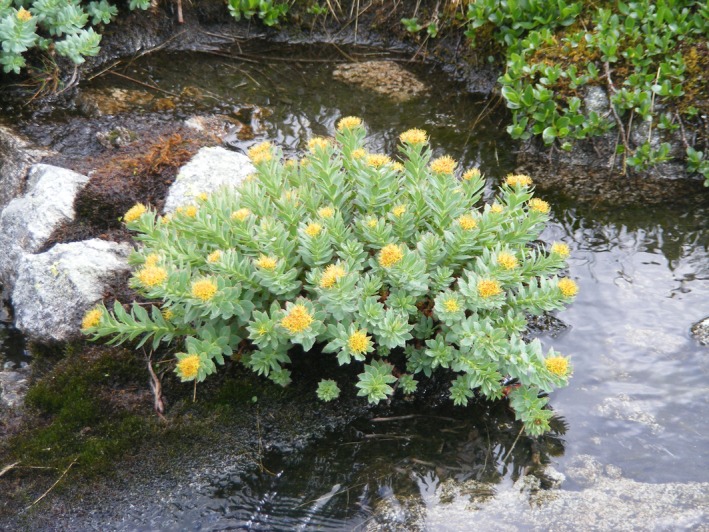
*Rhodiola rosea* L.

The number of species in the *Rhodiola* genus varies according to different authors between 60 (Ohba, 2005) to 200 (Germano & Ramazanov, 1999). This genus (fam. Crassulaceae) is one of the most studied plant genera of QTP origin (Mayuzumi & Ohba, 2004; Zhang et al., 2014). Phylogenetic studies have revealed a relatively rapid diversification and radiation with high species diversity on the QTP and adjacent regions. Only five species are distributed in northeast Asia and three species of *Rhodiola* exhibit intercontinental distribution. These are *Rhodiola integrifolia* Raf. that occurs on both sides of the Bering Strait, *Rhodiola rhodanta* (A. Gray) H. Jacobsen that is distributed only in the western part of North America (Ohba, 1989) and *R. rosea*. L. with the broadest range among all *Rhodiola* species, ranging from East Asia, toward the Arctic regions, Europe as well as eastern North America. In Europe *R. rosea* is distributed in Iceland, the British Isles, the European Alpine System, and Scandinavia (Hegi, 1963). It is a dioecious, cold‐adapted perennial, occupying a narrow range of the arctic‐alpine habitats (Figure 1). In eastern North America, it is restricted to the eastern coastal areas (Cuerrier, Archambault, Rapinski, & Bruneau, 2015; Ohba, 1989; Olfelt & Freyman, 2014).

Due to its extremely high morphological variability as described by Ohba (1981), there are several taxonomic interpretations within *R. rosea*. Recently, the Plant List reincludes this taxa in the genus *Sedum*, the valid name considered to be *Sedum roseum* (L.) Scop. (Plant List https://www.theplantlist.org). The Flora of China differentiates two varieties, *R. rosea* var. *rosea* with bigger leaves, longer flowering stem and almost entire leaf margin while *R. rosea* var. *microphylla *has smaller leaves, shorter flowering stem and the leaves are serrate (Fu & Ohba, 2001). Gontcharova, Gontcharov, Yakubov, and Kondo (2009) recognize three subspecies: *R. rosea* subsp. *rosea*, *R. rosea* subsp. *Arctica*, and *R. rosea* subsp. *sachalinensis *based on seed surface morphology. Yanbaev, Bairamgulov, Redkina, and Mullagulov (2007) reported *Rhodiola iremelica* Boriss. as an endemic species to the southern Urals, but based on molecular evidences, using the internal transcribed spacer György, Szabó, Bacharov, and Pedryc (2012) could not differentiate this species proposing this taxa only at subspecies rank of *R. rosea*. Several other molecular studies also demonstrate the high genetic variability within this taxon (Elameen, Klemsdal, Dragland, Fjellheim, & Rognli, 2008; György, Fjelldal, Szabo, Aspholm, & Pedryc, 2013; György, Vouillamoz, Ladányi, & Pedryc, 2014; Kozyrenko, Gontcharova, & Gontcharov, 2011).

Zhang et al. (2014) stated that *Rhodiola* species of disjunct intercontinental distribution have reached North America at least twice, via Beringia and via the amphi‐atlantic route. Latter route seems to fit well for *R. rosea* with its extremely small, winged seeds compared to other species. This particular seed morphology makes *R. rosea* a candidate species for long‐distance dispersal that most probably dates to the period when the NALB (Northern Atlantic Land Bridge) was not available anymore for species’ migration.

Molecular study applied on the European populations of *R. rosea* by microsatellite markers has revealed distinct clusters of populations within the EAS and suggested at least two glacial refugia for the species, one in the area of the Eastern Alps and the Carpathians and another one in the Dolomites (György, Vouillamoz, & Höhn, 2016).

With its wide and disjunct circumboreal distribution and high genetic variation, *R. rosea* seems to be a model plant species to study spatial diversification and migration from the QTP toward the Northern Hemisphere. Kozyrenko et al. (2011) based on ISSR markers, have come to the conclusion that at least two distinct evolutionary lines exist within *R. rosea*. The species migrated from its center of origin westward along the Ural Mts. and eastward by the mountain ridges of eastern Siberia. In a recent study on the chloroplast *trn* L–F region, Cuerrier et al. (2015) reported the existence of two infraspecific variants. Coastal and Alpine populations were found to differ in sharing two indels (duplication of 23 and 19 bp) suggesting for coastal population a common North American and Scandinavian origin, while the Alpine populations seem to have originated from Eurasia via the Central Asian highland corridor.

Accordingly, we assumed that along the westward expansion of the species, *R. rosea* has diversified within the area of Europe (EAS), which resulted in the two infraspecific variations observed by Cuerrier et al. (2015). These genetic lineages potentially contributed to the colonization of the Northern American continent. In the present study, chloroplast sequence data were used to gain further insights into the species’ genetic diversity along its distribution range, especially in Europe and by using a phylogeographic approach to propose a historical biogeographic scenario for the present‐day distribution of the species, focusing on the genetic lineages and evolution within the EAS.

## 2 MATERIALS AND METHODS

### 2.1 Sampling material

Altogether 44 populations of *R. rosea* were sampled, which are listed in Table 1. Mostly leaves were collected and stored at −20ºC. In some cases, seeds were obtained from Pharmaplant Ltd., germinated and the plantlets were used as source of DNA. Vouchers of the samples were deposited at Szent István University. DNA was extracted with SP Plant Mini Kit (Omega, VWR International Kft, Budapest). DNA concentration and quality was assessed using NanoDrop (BioScience, Hungary) and visually checked on 1% agarose gel.

**Table 1:** The studied *Rhodiola rosea* localities and the accession numbers of the sequences deposited in the GenBank

Site	Country	Origin or GPS co‐ordinates	GenBank accession nr. trnL‐F	GenBank accession nr. psbA5’R‐matk8F	GenBank accession nr. psbA‐trnH	GenBank accession nr. psbB‐psbH	GenBank accession nr. 5’rpS12‐rpL20	GenBank accession nr. trnCF‐ycf6R
Akureyri	Iceland	Botanical garden of Univ. of Oulu	KX078522	MG938069	MG938118	MG938169	MG938207	MG938246
Kilpisjarvi	Finland	Bertalan Galambosi, MTT, Finland	KX078539	MG938070	MG938112	MG938166	MG938203	MG938255
Halti	Finland	Bertalan Galambosi, MTT, Finland	KX078536	MG938071	MG938145	MG938184	MG938202	MG938257
Varanger fjord	Norway	Paul Erik Aspholm, Bioforsk, Norway	KX078561	MG938072	MG938113	MG938167	MG938204	MG938253
Kvaloya	Norway	Paul Erik Aspholm, Bioforsk, Norway	KX078542	MG938073	MG938115	MG938171	MG938205	MG938254
Sligo	Ireland	Andreas Plescher, Pharmaplant, Germany	KX078552	MG938074	MG938147	MG938174	MG938237	MG938251
Dingel	Ireland	Andreas Plescher, Pharmaplant, Germany	KX078530	MG938106	MG938134	MG938173	MG938236	MG938242
Inishmore	Ireland	53°07′19.22″N, 9°45′05.61″W	KX611154	MG938076	MG938136	MG938175	MG938208	MG938244
Cadair Idris	Great Britain	52°41′39.61″N, 3°54′28.31″W	KX078562	MG938075	MG938137	MG938168	MG938206	MG938245
Novaya Zemlya	Russia	Dmitry Bacharov, Komi Science Center, Russia	KX078547	MG938077	MG938144	MG938176	MG938200	MG938262
Kola peninsula	Russia	Botanical garden of Univ. of Oulu	KX078541	MG938081	MG938146	MG938170	MG938212	MG938256
Tsukts peninsula	Russia	Botanical garden of Univ. of Oulu	KX078557	MG938084	MG938122	MG938165	MG938214	MG938243
Anadir	Russia	Botanical garden of Univ. of Oulu	KX078524	MG938086	MG938119	MG938177	MG938239	MG938283
North‐Ural	Russia	Botanical garden of University of Oulu	KX078546	MG938078	MG938135	MG938162	MG938209	MG938260
Yakutia	Russia	Andreas Plescher, Pharmaplant, Germany	KX078563	MG938103	MG938151	MG938178	MG938226	MG938263
Kokorja Altai	Russia	Andreas Plescher, Pharmaplant, Germany	KX078540	MG938089	MG938150	MG938172	MG938216	MG938261
Tara Altai	Russia	Andreas Plescher, Pharmaplant, Germany	KX078553	MG938088	MG938149	MG938191	MG938215	MG938259
Ala Archa	Kyrgystan	42°29′48.12″N, 74°28′48.76″E	KX078523	MG938091	MG938148	MG938181	MG938238	MG938249
Rila	Bulgaria	Andreas Plescher, Pharmaplant, Germany	KX078551	MG938090	MG938121	MG938179	MG938213	MG938281
Triglav	Slovenia	46°19′6.87″N, 13°50′32.85″E	KX078556	MG938094	MG938125	MG938192	MG938220	MG938274
Passo Gavia	Italy	46°20′18.50″N, 10°29′17.20″E	KX078548	MG938068	MG938143	MG938190	MG938221	MG938277
Val Fredda	Italy	45°55′23.20″N, 10°23′51.04″E	KX078559	MG938092	MG938131	MG938194	MG938234	MG938275
Col Bricon	Italy	46°16′48.73″N, 11°45′27.34″E	KX078529	MG938064	MG938142	MG938153	MG938222	MG938278
Erdemolo	Italy	46°06′40.60″N, 11°22′38.29″E	KX078532	MG938067	MG938123	MG938156	MG938224	MG938279
Tonale	Italy	46°14′23.44″N, 10°34′52.31″E	KX078555	MG938065	MG938130	MG938193	MG938223	MG938276
Fedaia	Italy	46°27′29.27″N, 11°51′53.03″E	KX078534	MG938066	MG938124	MG938183	MG938225	MG938280
Prabichl	Austria	47°30′58.79″N, 14°56′53.77″E	KX078550	MG938097	MG938129	MG938189	MG938232	MG938270
Zirbitzkogel	Austria	47°03′46.01″N, 14°33′57.62″E	KX078564	MG938100	MG938141	MG938195	MG938227	MG938271
Hochkar	Austria	47°43′20.47″N, 14°55′24.56″E	KX078537	MG938102	MG938109	MG938185	MG938231	MG938273
Dürrenstein	Austria	47°47′22.57″N, 15°04′10.85″E	KX078531	MG938101	MG938108	MG938182	MG938219	MG938272
Triglav	Slovenia	46°18′51.29″N, 13°47′10.37″E	KX078556	MG938094	MG938125	MG938192	MG938220	MG938274
Binntal	Switzerland	José Vouillamoz, Agroscope, Switzerland	KX078525	MG938095	MG938132	MG938152	MG938228	MG938268
Unteralp	Switzerland	José Vouillamoz, Agroscope, Switzerland	KX078558	MG938104	MG938133	MG938155	MG938233	MG938265
Mattmark	Switzerland	José Vouillamoz, Agroscope, Switzerland	KX078544	MG938098	MG938126	MG938186	MG938230	MG938269
Val de Nomnom	Switzerland	José Vouillamoz, Agroscope, Switzerland	KX078560	MG938096	MG938127	MG938154	MG938235	MG938266
Piano dei Canali	Switzerland	José Vouillamoz, Agroscope, Switzerland	KX078549	MG938099	MG938128	MG938188	MG938229	MG938267
Mengusovska dolina	Slovakia	49°10′28.23″N, 20°03′27.24″E	KX078545	MG938083	MG938140	MG938187	MG938198	MG938264
Chopok	Slovakia	48°56′43.27″N, 19°36′45.70″E	KX078527	MG938082	MG938138	MG938157	MG938197	MG938247
Lomnicky stit	Slovakia	49°12′20″N, 20°13′31″E	KX078543	MG938105	MG938139	MG938158	MG938196	MG938248
Fagaras	Romania	45°36′6.09″N, 24°36′59.33″E	KX078533	MG938107	MG938117	MG938161	MG938201	MG938252
Cindrel	Romania	45°34′32.58″N, 23°45′48.10″E	KX078528	MG938085	MG938120	MG938163	MG938218	MG938240
Calimani	Romania	47°07′13′′N 25°10′17′′E	KX078526	MG938087	MG938111	MG938164	MG938217	MG938250
Grau Roig	Andorra	42°31′26.81″N, 1°41′19.92″E	KX078535	MG938079	MG938114	MG938160	MG938210	MG938241
Juclar	Andorra	42°35′54.11″N, 1°41′57.16″E	KX078538	MG938080	MG938116	MG938159	MG938211	MG938258
Tavargatai	Mongolia	Andreas Plescher, Pharmaplant, Germany	KX078554	MG938093	MG938110	MG938180	MG938199	MG938282

### 2.2 PCR amplification of the studied chloroplast regions

After preliminary screening of twelve regions of the chloroplast genome, the following six regions proved to amplify satisfactorily: *trnL‐trnF*, *psbA‐3’trnK‐matK*, *psbA‐trnH*, *psbB‐psbH*, *5′rpS12‐rpL20*, and *trnC‐ycf6* (Shaw et al., 2005).

Amplifications were performed in 25 μl reaction volume containing 20–80 ng DNA, 10× PCR reaction buffer, 2.5 mM MgCl_2_, 0.02 mM dNTP mix, 2.5 μmol of each 5′ and 3′ end primers, 1 unit of DreamTaq DNA polymerase (Fermentas, Waltham, MA, USA), 2% BSA and 1% DMSO and sterile distilled water. PCR was carried out in a Swift MaxPro thermocycler (Esco Healthcare Pte, Singapore) as described by Taberlet, Gielly, Pautou, and Bouvet (1991), annealing temperature of each primer pair is shown in Table 2. The PCR products were loaded on a 1% (w/v) ethidium bromide‐stained agarose gel in 1×TBE buffer with xylencyanol loading buffer to verify the amplification. Fragment sizes were estimated by comparison with the 1 kb DNA ladder (Fermentas, Waltham, MA, USA). The amplified fragments were purified using CleanSweep PCR purification kit (Thermo Fisher Scientific, Waltham, MA, USA) for direct sequencing. In a few cases, two or three fragments were amplified, which were cleaned with the EZ‐10 Spin Column DNA Gel Extraction Kit (Bio Basic Canada Inc., Markham, ON, Canada), cloned into pTZ57R/T vector (InsTAclone PCR Cloning Kit, Thermo Fisher Scientific, Waltham, MA, USA) and transferred into DH5α competent cells. Plasmid DNA was isolated with the EZ‐10 Spin Column Plasmid DNA Minipreps Kit (Bio Basic Canada Inc., Markham, ON, Canada). Sequencing was performed in an automated sequencer ABI PRISM 3100 Genetic Analyser (Applied Biosystems, Foster City, CA, USA). For each fragment, the nucleotide sequences were determined in both directions. Forward and reverse sequences were edited and assembled using MEGA7 (Kumar, Stecher, & Tamura, 2016). DNA sequences were compared using BLASTN at NCBI and alignments were built with ClustalW in Bioedit 7.2.5 (Hall, 1999; Thomson, Higgins, & Gibson, 1994). All sequences have been deposited in GenBank (KX078522–64, KX611154, and MG938064–MG938283).

**Table 2:** Summary of the genetic diversity indices estimated for the six chloroplast regions studied

Locus/Region	Primers	Length (bp)	Indels	SNPs	*D*	PIC	*π*	Nh	Hd
*trnL‐trnF*	*5*′*trnL* *^CAA^* *−trnFGAA*	921–1,055	5	10	0.1161	0.603	0.002070	7	0.7696
*psbA* *−3*′*trnK‐matK*	*matk8F‐ psbA5*′*R*	846–854	6	23	0.7468	0.800	0.005340	18	0.9050
*psbA‐trnH*	*psbA* *−trnHGUG*	374–417	13	30	0.6395	0.816	0.027810	27	0.9460
*psbB‐psbH*	*psbB* *−psbH*	634–653	5	8	−1.1590	0.370	0.001690	8	0.6560
*5*′*rpS12‐rpL20*	*5*′*rpS12−rpL20*	911–930	4	18	1.3508	0.900	0.003670	9	0.7620
*trnC‐ycf6*	*trnC* *^GCA^* *F* *−ycf6R*	892–900	2	22	0.8610	0.630	0.005550	14	0.8640
Mean		–	–	–	0.4259	0.6865	0.007688	13.83	0.8171

Note

*D*: Tajima's *D* value; Hd: haplotype diversity; Nh: number of haplotypes; PIC: polymorphic information content; *π*: Nucleotide diversity.

In some samples, not one, but two, (Triglav, Passo Gavia, Val Fredda, Zirbitzkogel, Präbichl) or even three fragments (Hochkar) were detected on the agarose gel in case of the *trnL‐F* locus. After cloning and sequencing, these fragments all turned out to represent the same sequence. Duplication has happened in the primer binding site and due to this one (or two) of the fragments was an artifact.

### 2.3 Diversity and differentiation analysis

Genetic diversity for each loci, that is, the number of haplotypes (*h*), haplotype diversity (Hd) (Nei & Tajima, 1981), and nucleotide diversity (*p*) (Nei, 1987) were calculated using the software DnaSP v5.1.0.1 (Librado & Rozas, 2009). Genetic distances (*p*‐uncorrected) within and between lineages were calculated with MEGA7 (Kumar et al., 2016). Polymorphic information content (PIC) values were calculated for each chloroplast locus based on the formula: PIC = 1 − ∑(*P_ij_*)2, where *P_ij_* is the frequency of the *i*th allele revealed by the *j*th marker summed across all alleles amplified (Botstein, White, Skolnick, & Davis, 1980). The marker is highly informative if PIC value is >0.5 but only reasonably informative if PIC value is ranging from 0.25 to 0.5.

PopART (Leigh & Bryant, 2015) with implemented Templeton‐Crandall‐Singh (TCS) statistical parsimony network analysis (Clement, Snell, Walker, Posada, & Crandall, 2002) was used to evaluate genealogical relationships among sequences. Each insertion/deletion (indel) was considered as a single mutation event, and all indels were therefore coded as single positions in the final alignments. The connection limit for the TCS analysis was 95% and gaps were treated as a fifth state.

The Bayesian clustering approach implemented in STRUCTURE 2.3.4 (Pritchard, Stephens, & Donnelly, 2000) was used to infer groups or subpopulations in the sequence dataset. The analysis was performed with an admixture linkage model with correlated allele frequencies. K value (the number of genetic groups) was set to 1–10 with a burn‐in period of 105 steps followed by 106 repetitions of Markov chain Monte Carlo (MCMC), which is capable to deal with linked markers. Twenty repetitions were set for each run. The web‐based STRUCTURE HARVESTER (Earl & von Holdt, 2012) was used to apply the Evanno method (Evanno, Regnaut, & Goudet, 2005) to detect the value of the optimal *K* that best fit the sequence data. The 20 simulations were averaged using CLUMPP v1.1.2 (Jakobsson & Rosenberg, 2007) and represented in the form of bar graphs using POPHELPER (Francis, 2017).

### 2.4 Phylogenetic analysis

The *trnL‐F* region was evaluated for phylogenetic construction, since GenBank (NCBI) sequences for other geographical regions, including Greenland and North America, were only available for this locus. Prior the analysis, FastGap 1.2 (Borchsenius, 2009) was used to code the phylogenetic information of gaps (indels) as binary characters within the sequence matrix by applying the “simple indel coding” algorithm of Simmons and Ochoterena (2000). The refined alignment of sequences and the indel binary matrix was incorporated in the concatenated dataset. The maximum likelihood (ML) approach (Felsenstein, 1981) implemented in RaxMLGUI 1.5b2 (Silvestro & Michalak, 2012) was used for tree construction, which is based on explicit models of sequence evolution and proved to be statistically robust. RaxMLGUI was run under the GTR nucleotide substitution model with gamma‐distributed rate heterogeneity (GTR+Γ). Bootstrap support of branches was calculated with 1,000 data resamples. *Rhodiola semenovii*, *Rhodiola kirilowii*, and *R. integrifolia* voucher specimens were used as outgroups in the phylogenetic analyses.

## 3 RESULTS

We sequenced six loci of the chloroplast genome of *R. rosea* in a collection of 44 sites from Eurasia. The six regions covered 4,943 bases. Within our alignment, we observed 111 nucleotide substitutions (of which 52 were singletons) and 37 indels of which 25 were duplications. Two of the six loci were earlier used in other studies with *Rhodiola* species (*trnL‐F* by Cuerrier et al., 2015 and *psbA‐trnH^GUG^* by Zhang et al., 2014). The mean nucleotide diversity estimates for these was *π* = 7.688 × 10^−3^. Tajima's *D* values for all loci studied did not show a significant deviation from neutrality (Table 2).

We assigned the studied genotypes to five geographic regions: Asia, the Carpathians (including the Rila Mt. from the mountain region of the Balkan), the Alps (including the Pyrenees), the British Isles, and Scandinavia. Among the chloroplast markers, *psbA‐trnH^GUG^* had the highest levels of nucleotide diversity across Eurasia. The mean nucleotide diversity level was 27.810 × 10^−3^, which is a magnitude higher than for the rest of the markers. PIC value, nucleotide diversity, number of haplotypes and haplotype diversity are compiled for all cpDNA regions in Table 2.

### 3.1 Analysis of the studied loci

In case of the *trnL‐F* locus, the amplified fragment length was between 921 and 1,055 bp resulting in an alignment of 1,109 bp length.

**Figure 2:**
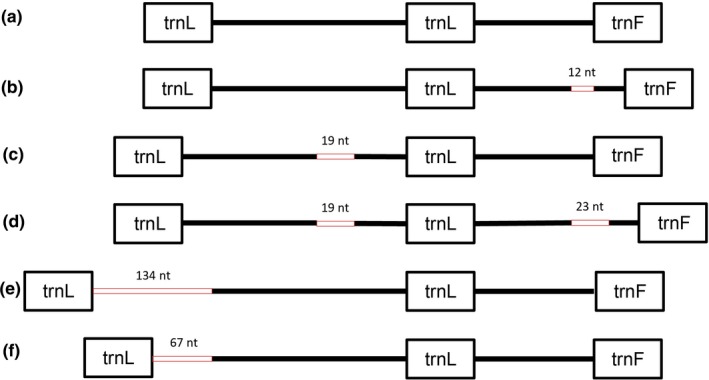
The structure of the studied *trnL‐F* region. Altogether six versions of this sequence have been detected. (a) is without any insertion, (b) contains one 12 bp insertion close to the 3′ end of the second intron, (c) contains a 19 bp insertion in the first intron, which was previously described by Cuerrier et al. (2015), (d) contains a 19 bp insertion in the first intron and a 23 bp insertion in the second intron both described previously by Cuerrier et al. (2015), (e) contains a 134 bp intron right at the 5′ end of the first intron and (f) contains a 67 bp insertion also at the 5′ end of the first intron

Altogether five indels were identified at four sites of the *trnL‐F* region and ten SNP, of which seven are singletons. Indels of 23 and 19 bp were already known from this region (Cuerrier et al., 2015), which were also detected in this study. Both are duplications. Furthermore, an indel of 12 bp was found close to the 3′ end, which is also a duplication. At the 5′ end, an insertion of 67 bp was detected alone or duplicated (Figure 2).

In case of the *psbA5’R‐matk8F* locus, the amplified fragment length was between 845 and 852 bp resulting in an alignment of 863 bp length. This region contained six indels of short sizes at four sites and 23 SNPs of which more than 50% were singleton. The region of *psbA‐trnH^GUG^* was found to be extremely variable. The amplified fragment length was between 320 and 361 bp resulting in an alignment of 393 bp length. Compared to the small fragment size, 12 indels were detected of varying sizes at nine sites. Also many, 30 SNPs were found of which only six were singletons. In case of the *psbB‐psbH* locus, the amplified fragment length was between 634 and 653 bp resulting in an alignment of 682 bp length. The region contained five indels at four sites ranging between four and 19 bp and eight SNPs of which six were singletons. The amplified fragment length of the *5’rpS12‐rpL20* locus was between 911 and 930 bp and the aligned length was 937 bp. The region contained six indels of small size at four sites and 18 SNPs most of which were singletons. Finally, in case of the *trnC^GCA^F‐ycf6R* locus, the amplified fragment length was between 892 and 900 bp resulting in an alignment of 900 bp length. The region contained just two small sized indels at two sites and 22 SNPs of which seven were singletons. All sequences are deposited in the NCBI GenBank under the accession number of KX078522–64, KX611154, and MG938064–MG938283 (Table 1).

### 3.2 Haplotype network analysis

**Figure 3:**
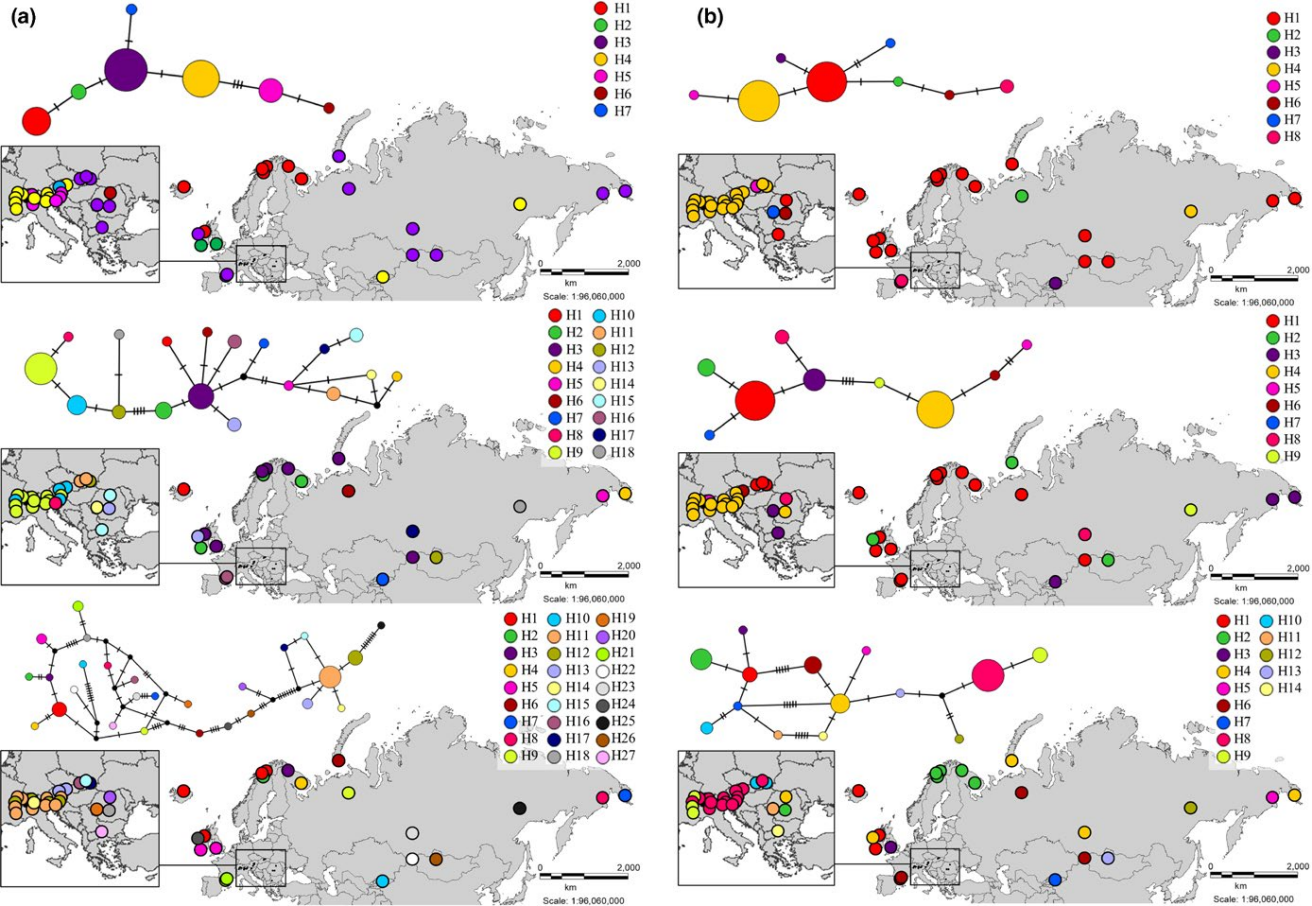
Haplotype networks obtained from the Templeton‐Crandall‐Singh analysis of the studied 44 *Rhodiola rosea* samples. The size of the circle represents the frequency of each haplotype. (a) A: trnL‐F, B: psbA5'R−matk8F, C: psbA−trnHGUG, (b) D: psbB−psbH, E: 5'rpS12−rpL20, F: trnCGCAF−ycf6R. Different colors correspond to the different samples. Black dots indicate missing intermediate haplotypes that were unobserved in the analyzed sample set. Hash marks on branches represent mutation steps (number of base pair changes) between haplotypes

There are between seven and 27 haplotypes for each loci, separately in our samples, and we constructed the haplotype networks for each of these loci. Unrooted haplotype genealogies were estimated from the substitution polymorphisms (including indels coded as single characters) observed at each of the six loci (Figure 3), and each analysis yielded one most parsimonious arrangement.

**Figure 4:**
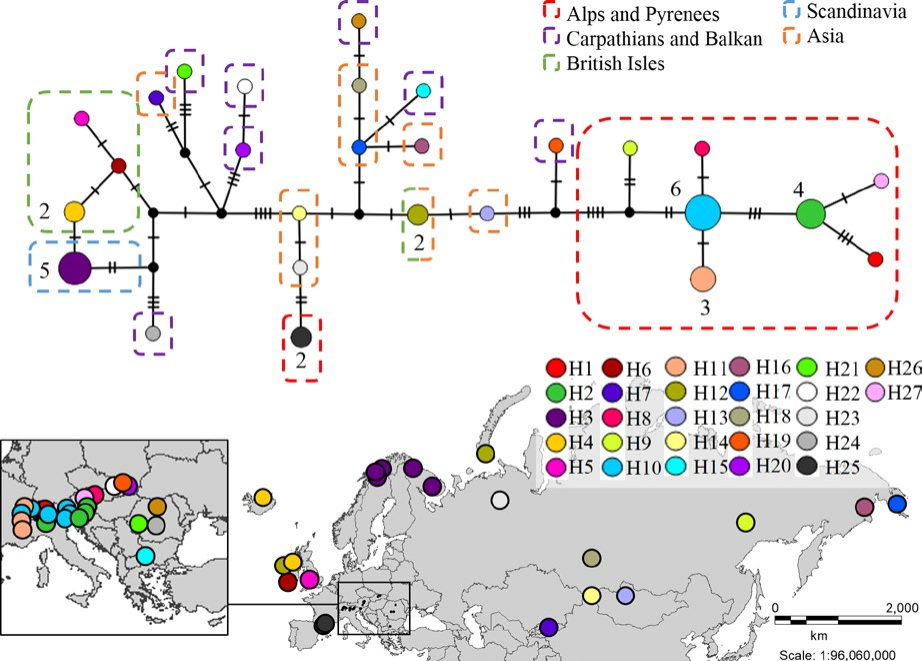
Concatenated haplotype network obtained from the Templeton‐Crandall‐Singh analysis of the studied 44 samples based on four chloroplast regions: trnL‐F, psbB−psbH, 5'rpS12−rpL20, trnC−ycf6. The size of the circle represents the frequency of each haplotype. Different colors correspond to the different haplotypes. Black dots indicate missing intermediate haplotypes that were unobserved in the analyzed sample set. Hash marks on branches represent mutation steps (number of base pair changes) between haplotypes. Colored frames represent the five geographical regions we assigned the samples in

There is a phylogeographic pattern in the distribution of *R. rosea* haplotypes, which provides clues about the spread of roseroot across Eurasia. For all six loci studied, the samples from Asia are in the center of the network, representing the origin of the species. Samples from the Alps are in a clearly separate haplotype cluster, with a single link to Asian samples. The samples from the Carpathians are clustered together with the Asian samples or are in direct connection with those. Scandinavian and British Isles samples are connected to each other and are connected to the Asian samples, sometimes sharing the haplotype. For *trnL‐trnF*, *psbB‐psbH*, and *5’rpS12‐rpL20*, a rather simple network structure outlined, while for *psbA5’R‐matk8F*, *psbA‐trnH^GUG^*, and *trnC^GCA^‐ycf6R*, the structure is more complex, but still biogeographically interpretable. A concatenated haplotype network (Figure 4) was also built, but since using all six loci resulted in a very complex network, in which almost all samples represented a separate haplotype, we decided to omit the two most polymorphic loci (*psbA5’R‐matk8F*, *psbA‐trnH^GUG^*).

**Figure 5:**
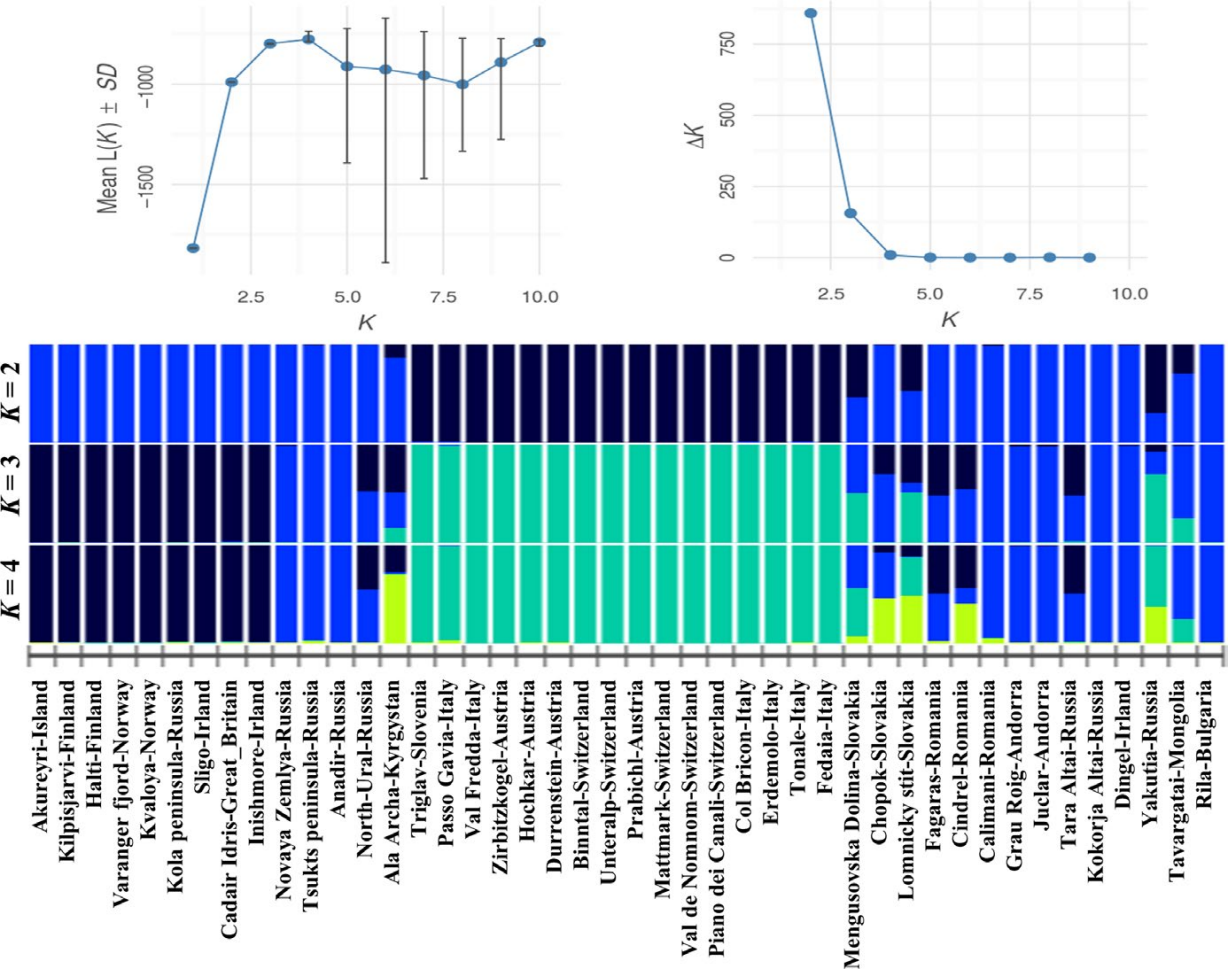
Estimated structure for *K* = 2, *K* = 3 and *K* = 4 of assignment analysis performed in STRUCTURE (Pritchard et al., 2000). The most likely number of clusters (*K*) detected by Evanno et al. (2005) method implemented in STRUCTURE HARVESTER (Earl & von Holdt, 2012). Each individual is represented by a vertical stacked column indicating the proportions of *K *groups

We used Bayesian Structure analysis to see whether the samples from the same geographic region are clustered together in the same group. Even though we assigned the samples into five geographical groups, the Evanno method indicates that a *K* = 3 model fits best the data (Figure 5) or less likely a *K* = 4 model. When three groups are formed, Atlantic and Scandinavian samples (Finnland, Norway, Iceland, British Isles, Kola Peninsula) clustered together. Another obvious group is formed by samples from the Alps and northern parts of the Carpathians. The rest of the samples, forming the third group, are more admixed including samples from Asia, the Pyrenees and some part of the Carpathians. When *K* = 4 is considered, besides the group of the Atlantic and Scandinavian samples and the Alpine samples two more admixed groups are formed both including Asian and Carpathian samples.

Since there are data available in the literature only for *trnL‐F* and *psbA‐trnH^GUG^* loci in case of *R. rosea*, these results provide the opportunity of a much more comprehensive evaluation. In case of the *trnL‐F* locus, seven haplotypes were distinguished, which are in accordance with the presence or absence of the different indel. Cluster A (Figure 2; including also one SNP) contains Eurasian samples including samples from the EAS, all Asian samples and one of the Irish samples. None of these sequences contain any indels. Cluster B contains one single sample from the Calimani Mt. (Eastern‐Carpathians), which is characterized by a duplication of 12 bp close to the 3′ end of the *trnL‐trnF* region. Cluster C includes one sample from Wales and one from Ireland both bearing only one of the indels that was previously described by Cuerrier et al. (2015). Cluster D contains samples from the Atlantic Coast, Northern Finland, Northern Norway, Kola‐peninsula, Iceland, and also an Irish sample. These samples contain both indels that were previously described by Cuerrier et al. (2015). Cluster E includes a single sample from Hochkar (Göstling Alps) and F includes some more samples from the Austrian Alps, Julian Alps, and the Dolomites. These latter two groups are closely related, not including the two earlier described indels by Cuerrier et al. (2015), but another in the 5′ end of the locus. They both have a 67 bp duplication, which generated the double band on the agarose gel and cluster E has besides this duplication a further 54 bp insertion, which is partly a duplication of the other 67 bp insertion, which generated the triple band on the agarose gel. Blast analysis revealed that this 67 bps have not been detected earlier in *R. rosea*. 53 bases out of these 67 are present in the *trnL‐trnF* region of *Rhodiola dumulosa*, *Rhodiola yunnanensis*, and *Rhodiola cretinii*. The unmatched bases are 5′‐AAAAAAAGGGGGGG‐3′.

**Figure 6:**
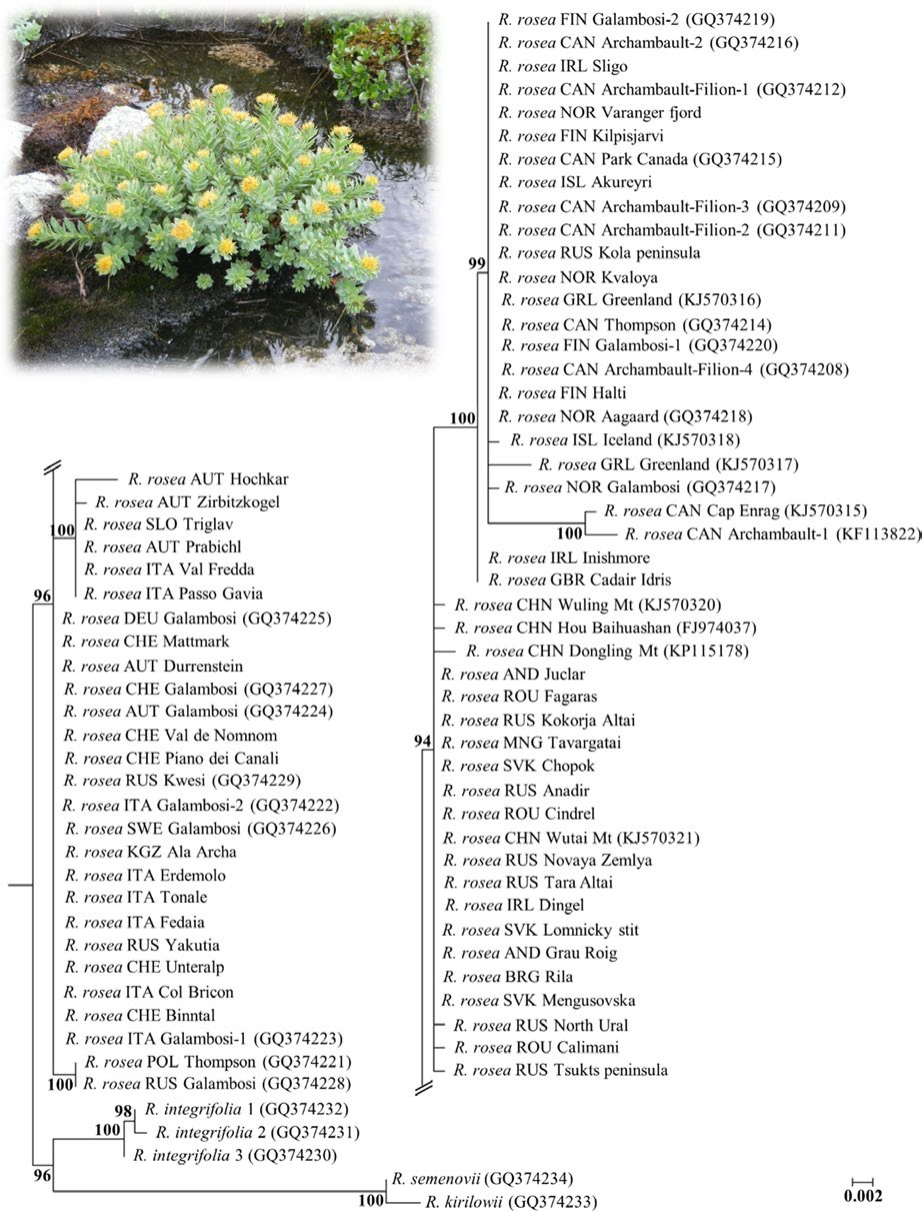
Maximum likelihood (ML) tree with support values (1,000 data resample) based on the *trnL‐F* region of the studied 44 *Rhodiola rosea* samples and other 34 sequences retrieved from the NCBI GenBank database. Tree was generated using *Rhodiola integrifolia*,* Rhodiola semenovii*, and *Rhodiola kirilowii* as outgroup

In case of the *trnL‐F* loci, sequences from other geographical regions are available in the GenBank, like Greenland, Canada, China, and also some European countries. Figure 6 shows a maximum likelihood tree derived from the combined matrix of our sequences supplemented with other *R. rosea* and also *R. integrifolia*,* R. kirilowii*,* R. semenovii trnL‐trnF *sequences mined from the GenBank. In these analyses, sequences were modified, gaps (indels) were coded as single mutations in order to weight SNPs and indels equally. Most of the samples from the Alps, from the Carpathians and from Asia are clustered together including also other *Rhodiola* species. Based on the presence of the long indels, six samples from the Alps are distinguished and also based on the presence of the two previously known indels the Scandinavian, North American, and British Isle samples are forming a separate clade.

A few sequences are also available for the *psbA‐trn* region in the GenBank. However, this locus proved to be the most variable region, so even though an attempt was made to generate a maximum parsimony tree also for these sequences a very complex tree was obtained without any clear clustering.

## 4 DISCUSSION

The noncoding regions of the chloroplast are widely used to study genetic lineages and relationships among taxa, and to describe complex historical biogeography of species and genera in relation to their present distribution. The main advantages of these chloroplast regions are the universality of the primers and the robustness of the amplification process (Taberlet et al., 2007).

In the study of DNA barcoding of *Rhodiola* species by Zhang et al. (2014, five frequently used sequences (*rbcL*, *matK*, *trnH*‐*psbA*, *ITS* and *trnL‐F*) were tested for their utility. Even though ITS sequences were found to be the most powerful, *trnL‐F* region showed to be also a promising alternative marker for barcoding *Rhodiola* species. Regarding phylogeographical patterns, the study by Cuerrier et al. (2015) has revealed two intraspecific variants of *R. rosea* based on this chloroplast region. Coastal and Alpine populations were found to differ in the presence or absence of two indels (duplications of 23 and 19 bp). Based on this, the authors have concluded that coastal populations of North America and Scandinavia are genetically separated from the Alpine populations. Our Structure analysis (Figure 5) and the extended maximum likelihood tree (Figure 6) confirm their findings, but on the other hand, our results revealed a more elaborated pattern within Europe. Both the Structure analysis (Figure 5) and the haplotype networks (Figures 3 and 4) identify the samples from the Central‐European mountains as a distinct group. In the QTP, which is considered to be the center of origin and in adjacent regions, no insertions are present in the *trnF‐L* region of roseroot. Neither were these insertions detected anywhere in Asia nor in most of the European samples. However, since there are some coastal samples in Europe like Dingel in Ireland, or Novaya Zemlya, Kamchatka in Asia which do not exhibit these two characteristic “coastal” indels (Cuerrier et al., 2015) and there are samples containing the two indels being not exactly nearby the coastal areas like Halti or Kilpisjärvi in Finland the term amphi‐Atlantic would be more accurate to be used instead of coastal.

**Figure 7:**
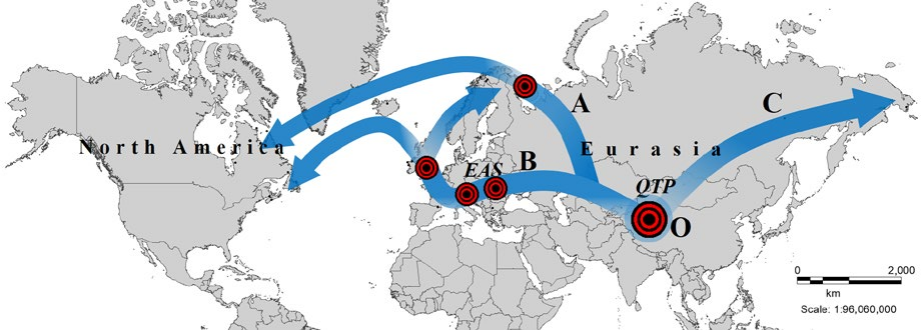
Hypothetical distribution routes for *Rhodiola rosea* based on *trn L‐F* sequences. A, two distinct ancient evolutionary lineages started from the QTP: an eastward and a westward expanding lineage. The westward route followed the mountain ranges of the Central Asiatic highland corridor and the EAS to the west and the Taymir‐Siberian region to the northwest. The mutations resulting in the two indels happened in the area of the British Isles in a stepwise manner and the altered lineage moved forward to the North to Scandinavia and to the western parts of North America; B, two distinct ancient evolutionary lineages started from the QTP: an eastward and a westward expanding lineage. The westward route followed the mountain ranges of the Central Asiatic highland corridor and the EAS to the west and the Taymir‐Siberian region to the northwest. The mutations resulting in the two indels have happened somewhere in the northern glacial refugia or even before the glaciations. The altered lineage has moved through the Amphi‐Atlantic route and in the British Isles it met the ancient lineage where they hybridized resulting the local unexpectedly high diversity. Signs are showing the mutation events

The lineage harboring the two insertions, which is distributed along the coastal parts of Scandinavia, the British Isles and in the eastern parts of North America suggest common origin of these populations. We interpret this pattern as evidence for the large periglacial distribution of *R. rosea* during the time of the Pleistocene from where it could have colonized the North Atlantic costs, Iceland, and North America postglacially (Abbott & Brochmann, 2003; Brochmann, Gabrielsen, Nordal, Landvik, & Elven, 2003; Schmitt, 2007). However, in the British Isles, both types are present (containing and missing the two insertions), while in Scandinavia only those with the two insertions were found. Moreover, we also observed individuals in Wales and in the Inismore island (Ireland) bearing only one insertion (Figure 3A). Accordingly, the earlier described two indels might have had their origin on the British Isles from where they have moved toward the north (Scandinavia) and west (North America; Figure 7B). Alternatively, individuals exhibiting the two insertions have expanded from an ancient, Taymir‐Siberian Northeastern lineage (Figure 7A) (Alsos et al., 2007) even before the onset of the glaciations (Taberlet et al., 1998) as it has been reported in some arctic species like *Saxifraga oppositifolia* (Holderegger & Abbott, 2003) or *V. uliginosum* (Alsos et al., 2005). In case of *V. uliginosum*, a deep phylogenetic split was presumed early before the glaciations. However, Atlantic cost seems to have genetic material of different origin. In case of *D. octopetala*, Scandinavian territories were considered to be even contact zones between the European and Eastern lineages (Skrede et al., 2006).

In *R. rosea*, the lineage without any insertion of the British Isles most probably originates from the EAS and underwent stepping stone mutation process on the British Isles. This is indicated by the existence of only one insertion present in Wales and in Inishmore island. Accordingly, the British Isles proved to be a major diversification center in case of *R. rosea*. Moreover, two other loci studied in our work, *psbA‐trnH^GUG^* and *trncGCA‐ycf6R*, support this hypothesis (Figure 3C,F).

Coastal and intercontinental colonization toward the northwest by long‐distance dispersal resulted in the eastern North American distribution of *R. rosea* as it has been earlier reported for other amphi‐arctic species (Alsos et al., 2015). Molecular analysis of *Rhodiola* species extending toward North America have reported the presence of the species at least since the Middle Pleistocene and suggested its entry into the American continent at least twice. According to Zhang et al. (2014), ancestors of *R. rhodantha* and *R. integrifolia* have reached the continent from the east via Beringia while *R. rosea* arrived more likely via the amphi‐Atlantic route, which is supported by our results as well.

Our *trnL‐F* results confirm the two distinct ancient evolutionary lineages from the QTP: an eastward and a westward expanding lineage, as proposed by Kozyrenko et al. (2011). After reaching the EAS most probably before the glacial cycles or at the beginning of the glacial cycles of the Pleistocene (Taberlet et al., 1998), *R. rosea* underwent a considerable diversification that resulted in a higher diversity in the Alpine‐Carpathian mountain ranges. In the Eastern Alps, (Julian Alps and the Austrian Alps) individuals forming the group E and F (based on *trnL‐F* haplotypes) do not have the insertions described formerly by Cuerrier et al. (2015) differentiating these samples from those distributed along the Atlantic coastal and intercontinental area. However, they do have a duplication of 67 bp in the 5′ end of the *trnL‐F* region. Group F includes beside the samples from the Eastern Alps also the samples from the Dolomites. Moreover, at the Hochkar (Austrian Alps) two insertions are present (beside the duplication a further 54 bp insertion, group E from Figure 2). This diversification might have dated back to the time of the glacial cycles of the Pleistocene when species were forced to migrate and underwent withdrawals and expansion events (Schönswetter et al., 2005; Taberlet et al., 1998). Also all other loci studied confirm diversification in the region of the EAS (Figures 3 and 4). Our former study based on nuclear microsatellites has already revealed that the Eastern Alps and the Dolomites exhibit a distinct genetic pattern compared to other Alpine regions and might have served as possible refugia for *R. rosea* (György et al., 2016) in the EAS. Indeed, several phylogeographical studies on alpine perennial plant species have mentioned the Eastern Alps and the surrounding lower mountainous regions as refugial territories, where species probably survived during the glacial cycles of the Pleistocene and could have recolonized the Alps in the postglacial (Mráz et al., 2007; Schönswetter et al., 2005; Tribsch & Schönswetter, 2003). The insertion detected in the *trnL‐F* region in the Hochkar sample (forming the group E, Figure 2) might represent an ancient lineage as it is also present in the northeastern and eastern Asian species, like *R. dumulosa*,* R. yunnanensis*,* R. cretinii*, and might date back to the time of the intensive diversification period of *Rhodiola* genus (Zhang et al., 2014). We can presume that the strong climate fluctuation of the Pleistocene might result also in the loss of some genetic material reaching the EAS formerly from the Central Asian highland corridor and have been only maintained in the refugial territory of the Eastern Alps.

Interestingly, in case of the *trnL‐F* region another, earlier not known duplication has been revealed within the EAS, namely in the sample of the Eastern Carpathians, the Calimani Mts. (Figure 3A). Although only one population was included in the study, we could detect the signs of a different gene stock only persisting in the Eastern Carpathians. Samples from the neighboring Southern Carpathians proved to be different as they belong to the large group without any insertions in the *trnL‐F* region (Figure 2, group A). The sample from the Calimani Mts. indeed represents a distinguished haplotype based on *psbA5’R‐matk8F*, *psbA‐trnH^GUG^*, and *5’rpS12‐rpL20* loci also. We consider it would be interesting to analyze more samples from this region to highlight the characteristics of the gene stock preserved along the Carpathians. Earlier, genetic lineages different from those of the Alps have been reported from the Northern Carpathians, the Tatra Mts.; in case of *D. octopetala*, an eastern genetic lineage was detected being more closely related to the northeast Russian ones than to the Alpine (Skrede et al., 2006). The available case studies reported also phylogeographical structuring of populations from the Eastern and Southern Carpathians (Mráz et al., 2007; Ronikier, 2011).

## 5 CONCLUSION

Our study explores high resolution in the genetic pattern of *R. rosea* a widely distributed arctic‐alpine perennial species of the Northern Hemisphere based on six chloroplast regions. As it has been already stated this species originates from the QTP from where it has expanded through the NH. Migration and diversification toward the east were documented by several studies (Hou & Lou, 2014; Kozyrenko et al., 2011). Based on the sequence alignment of the *trnL‐F* region, northern expansion toward Siberia and the colonization of western Eurasia have started most probably at the same historical time (Zhang et al., 2014). Our results support the migration of the species into Europe via the Central Asian highland corridor, reaching the EAS and also the western European edge, the British Isles. As it has been documented in earlier studies that glacial cycles of the Pleistocene have had strong influence on the genetic pattern of the arctic‐alpine species. In case of *R. rosea*, the EAS proved to be an important center of high genetic variation, especially the region of the Eastern Alps and the Dolomites where glacial refugia might have had existed. Although in many arctic‐alpine species postglacial colonization of northern Europe, Scandinavia was supposed to be from the EAS in case of *R. rosea* we only could detect a strong relation between the northern amphi‐Atlantic coastal parts and the British Isles. However, our former study based on microsatellite markers has already revealed the genetic differentiation of the Scandinavian populations from that of the EAS (György et al., 2016). The high variation and distinct genetic pattern preserved in the Alpine and Carpathian populations emphasizes the role of the EAS in the diversification of *R. rosea* most probably dating back to the glacial cycles of the Pleistocene and supporting the existence of long‐standing refugia. Apart from those of the EAS, a common lineage was detected along the Atlantic coast from the British Isles toward Scandinavia as well as Iceland and the Eastern parts of North America. Accordingly, the British Isles seems to represent the main link between the northern Atlantic and southern EAS lineages.

## AUTHORS’ CONTRIBUTIONS

ZG designed the research, collected the plant material, performed the molecular work, analyzed the sequence data, and wrote the manuscript, EGT participated in the collection of plant material, data interpretation and analysis and prepared the illustrations, NI performed part of the molecular work and analyzed the sequence data, BM participated in the collection of plant material and performed part of the molecular work, MH participated in the collection of plant material, discussed the results, and wrote the manuscript. All authors read and approved the final manuscript.

## DATA ACCESSIBILITY

DNA sequences: GenBank accessions (NCBI): KX078522–64, KX611154, and MG938064–MG938283.

## References

[ece34589-bib-0001] Abbott, R. , & Brochmann, C. (2003). History and evolution of the arctic flora: In the footsteps of Eric Hultén. Molecular Ecology, 12(2), 299–313. 10.1046/j.1365-294x.2003.01731.x 12535083

[ece34589-bib-0002] Abbott, R. J. , Smith, L. C. , & Milne, R. I. (2000). Molecular analysis of plant migration and Refugia in the Arctic. Science, 289, 1343–1346. 10.1126/science.289.5483.1343 10958779

[ece34589-bib-0003] Alsos, I. G. , Ehrich, D. , Eidesen, P. B. , Solstad, H. , Westergaard, K. B. , Schönswetter, P. , … Brochmann, C. (2015). Long‐distance plant dispersal to North. AoB Plants, 7, 19. 10.1093/aobpla/plv036 PMC4432000 25876627

[ece34589-bib-0004] Alsos, I. G. , Eidesen, P. B. , Ehrich, D. , Skrede, I. , Westergaard, K. B. , Jacobsen, G. H. , … Brochmann, C. (2007). Frequent long‐distance plant colonization in the changing arctic. Science, 316, 1606–1609. 10.1126/science.1139178 17569861

[ece34589-bib-0005] Alsos, I. G. , Engelskjøn, T. , Gielly, L. , Taberlet, P. , & Brochmann, C. (2005). Impact of ice ages on circumpolar molecular diversity: Insights from an ecological key species. Molecular Ecology, 14, 2739–2753. 10.1111/j.1365-294X.2005.02621.x 16029475

[ece34589-bib-0006] Borchsenius, F. (2009). *FastGap*,* Version 1.2* . Aarhus, Denmark: Department of Biological Sciences, University of Aarhus.

[ece34589-bib-0007] Botstein, D. , White, R. L. , Skolnick, M. , & Davis, R. W. (1980). Construction of a genetic linkage map in man using restriction fragment length polymorphisms. American Journal of Human Genetics, 32(3), 314. 6247908 PMC1686077

[ece34589-bib-0008] Brochmann, C. , Gabrielsen, M. T. , Nordal, I. , Landvik, J. Y. , & Elven, R. (2003). Glacial survival or tabula rasa? Taxon, 52, 417–450. 10.2307/3647444

[ece34589-bib-0009] Christe, C. , Caetano, S. , Aeschimann, D. , Kropf, M. , Diadema, K. , & Naciri, Y. (2014). The intraspecific genetic variability of siliceous and calcareous Gentiana species is shaped by contrasting demographic and re‐colonization processes. Molecular Phylogenetics and Evolution, 70, 323–336. 10.1016/j.ympev.2013.09.022 24099890

[ece34589-bib-0010] Clement, M. J. , Snell, Q. , Walker, P. , Posada, D. , & Crandall, K. A. (2002). TCS: Estimating gene genealogies. Parallel and Distributed Processing Symposium, International Proceedings, 2, 184 10.1109/ipdps.2002.1016585

[ece34589-bib-0011] Cuerrier, A. , Archambault, M. , Rapinski, M. , & Bruneau, A. (2015). Taxonomy of *Rhodiola rosea* L., with special attention to molecular analyses of Nunavik (Québec) populations In Cuerrier A., & Ampong‐Nyarko K. (Eds.), *Rhodiola rosea* (pp. 1–33). Boca Raton, FL: CRC Press Taylor and Francis Group.

[ece34589-bib-0012] Earl, D. A. , & von Holdt, B. M. (2012). STRUCTURE HARVESTER: A website and program for visualizing STRUCTURE output and implementing the Evanno method. Conservation Genetics Resources, 4(2), 359–361. 10.1007/s12686-011-9548-7

[ece34589-bib-0013] Elameen, A. , Klemsdal, S. S. , Dragland, S. , Fjellheim, S. , & Rognli, O. A. (2008). Genetic diversity in a germplasm collection of roseroot (*Rhodiola rosea*) in Norway studied by AFLP. Biochemical Systematics and Ecology, 36, 706–715. 10.1016/j.bse.2008.07.009

[ece34589-bib-0014] Evanno, G. , Regnaut, S. , & Goudet, J. (2005). Detecting the number of clusters of individuals using the software STRUCTURE: A simulation study. Molecular Ecology, 14(8), 2611–2620. 10.1111/j.1365-294X.2005.02553.x 15969739

[ece34589-bib-0015] Felsenstein, J. (1981). Evolutionary trees from DNA sequences: A maximum likelihood approach. Journal of Molecular Evolution, 17(6), 368–376. 10.1007/BF01734359 7288891

[ece34589-bib-0016] Francis, R. M. (2017). Pophelper: An R package and web app to analyse and visualize population structure. Molecular Ecology Resources, 17(1), 27–32. 10.1111/1755-0998.12509 26850166

[ece34589-bib-0017] Fu, K. T. , & Ohba, H. (2001). Crassulaceae In Wu Z. Y., & Raven P. H. (Eds.), Flora of China, Vol. 8 (pp. 202–268). Beijing, China: Science Press.

[ece34589-bib-0018] Germano, C. , & Ramazanov, Z. (1999). Arctic root (*Rhodiola rosea*): The powerful new ginseng alternative. New York, NY: Kensington Press.

[ece34589-bib-0019] Gontcharova, S. B. , Gontcharov, A. A. , Yakubov, V. V. , & Kondo, K. (2009). Seed surface morphology in some representatives of the genus *Rhodiola sect. Rhodiola* (Crassulaceae) in the Russian Far East. Flora, 204, 17–24. 10.1016/j.flora.2008.01.009

[ece34589-bib-0020] György, Z. , Fjelldal, E. , Szabo, A. , Aspholm, P. E. , & Pedryc, A. (2013). Genetic diversity of golden root (Rhodiola rosea L.) in northern Norway based on recently developed SSR markers. Turkish Journal of Biology, 37(6), 655–660. 10.3906/biy-1302-17

[ece34589-bib-0021] György, Z. , Szabó, M. , Bacharov, D. , & Pedryc, A. (2012). Genetic diversity within and among populations of roseroot (*Rhodiola rosea* L.) based on molecular markers. Notulae Botanicae Horti Agrobotanici Cluj‐Napoca, 40, 266–273. 10.15835/nbha4028212

[ece34589-bib-0022] György, Z. , Vouillamoz, J. F. , & Höhn, M. (2016). Microsatellite markers reveal common East Alpine‐Carpathian gene pool for the arctic–alpine *Rhodiola rosea* (Crassulaceae). Plant Systematics and Evolution, 302(6), 721–730. 10.1007/s00606-016-1302-x

[ece34589-bib-0023] György, Z. , Vouillamoz, J. F. , Ladányi, M. , & Pedryc, A. (2014). Genetic survey of *Rhodiola rosea* L. populations from the Swiss Alps based on SSR markers. Biochemical Systematics and Ecology, 54, 137–143. 10.1016/j.bse.2014.01.012

[ece34589-bib-0024] Hall, T. A. (1999). BioEdit: A user‐friendly biological sequence alignment editor and analysis program for Windows 95/98/NT. Nucleic Acids Symposium Series, 41(41), 95–98.

[ece34589-bib-0025] Hegi, G. (1963). Rhodiola, Rosenwurz In Hegi G. (ed.) Illustrierte Flora von Mitteleuropa, Band IV/2, Lieferung 2/3, zweite völlig neubearbeitete Edn (pp. 99–102). Hamburg/Berlin, GErmany: Paul Parey.

[ece34589-bib-0026] Holderegger, R. , & Abbott, R. J. (2003). Phylogeography of the arctic‐alpine *Saxifraga oppositifolia* (Saxifragaceae) and some related taxa based on cpDNA and its sequence variation. American Journal of Botany, 90(6), 931–936. 10.3732/ajb.90.6.931 21659189

[ece34589-bib-0027] Hou, Y. , & Lou, A. (2014). Phylogeographical patterns of an Alpine Plant, *Rhodiola dumulosa* (Crassulacant eae), inferred from chloroplast DNA sequences. Journal of Heredity, 105(1), 101–110. 10.1093/jhered/est072 24133162

[ece34589-bib-0028] Jakobsson, M. , & Rosenberg, N. A. (2007). CLUMPP: A cluster matching and permutation program for dealing with label switching and multimodality in analysis of population structure. Bioinformatics, 23(14), 1801–1806. 10.1093/bioinformatics/btm233 17485429

[ece34589-bib-0029] Kadereit, J. W. , Licht, W. , & Uhink, C. (2008). Asian relationships of the flora of the European Alps. Plant Ecology and Diversity, 1(2), 171–179. 10.1080/17550870802328751

[ece34589-bib-0030] Kozyrenko, M. , Gontcharova, S. B. , & Gontcharov, A. A. (2011). Analysis of the genetic structure of *Rhodiola rosea* (Crassulaceae) using inter‐simple sequence repeat (ISSR) polymorphisms. Flora, 206, 691–696. 10.1016/j.flora.2010.12.002

[ece34589-bib-0031] Kumar, S. , Stecher, G. , & Tamura, K. (2016). MEGA7: Molecular evolutionary genetics analysis version 7.0 for bigger datasets. Molecular Biology and Evolution, 33(7), 1870–1874. 10.1093/molbev/msw054 27004904 PMC8210823

[ece34589-bib-0032] Leigh, J. W. , & Bryant, D. (2015). Popart: Full‐feature software for haplotype network construction. Methods in Ecology and Evolution, 6(9), 1110–1116.

[ece34589-bib-0033] Librado, P. , & Rozas, J. (2009). DnaSP v5: A software for comprehensive analysis of DNA polymorphism data. Bioinformatics, 25(11), 1451–1452. 10.1093/bioinformatics/btp187 19346325

[ece34589-bib-0034] Mayuzumi, S. , & Ohba, H. (2004). The phylogenetic position of East Asian Sedoideae (Crassulaceae) inferred from chloroplast and nuclear DNA sequences. Systematic Botany, 29, 587–598. 10.1600/0363644041744329

[ece34589-bib-0035] Mráz, P. , Gaudeul, M. , Rioux, D. , Gielly, L. , Choler, P. , & Taberlet, P. (2007). Genetic structure of *Hypochaeris uniflora* (Asteraceae) suggests vicariance in the Carpathians and rapid post‐glacial colonization of the Alps from an eastern Alpine refugium. Journal of Biogeography, 34, 2100–2114. 10.1111/j.1365-2699.2007.01765.x

[ece34589-bib-0036] Nei, M. (1987). Molecular evolutionary genetics. New York, NY: Columbia University Press.

[ece34589-bib-0037] Nei, M. , & Tajima, F. (1981). DNA polymorphism detectable by restriction endonucleases. Genetics, 97(1), 145–163. 6266912 10.1093/genetics/97.1.145 PMC1214380

[ece34589-bib-0038] Ohba, H. (1981). A revision of Asiatic species of Sedoideae (Crassulaceae). Part 2. Rhodiola (subgen. Rhodiola, sect. Rhodiola). Journal of the Faculty of Science of the University of Tokyo, 13, 65–119, Section 3.

[ece34589-bib-0039] Ohba, H. (1989). Biogeography of the genus *Rhodiola* . Acta Phytotaxonomica Et Geobotanica, 38, 211–223.

[ece34589-bib-0040] Ohba, H. (2005). Rhodiola In Eggli U. (Ed.), Illustrated handbook of succulent plants: Crassulaceae, vol. 14 (pp. 210–227). New York, NY: Springer, Berlin‐Heidelberg, (2nd printing).

[ece34589-bib-0041] Olfelt, J. P. , & Freyman, W. A. (2014). Relationships of North American members of *Rhodiola* (Crassulaveae). Botany‐Botanique, 92, 1–10.

[ece34589-bib-0042] Pritchard, J. K. , Stephens, M. , & Donnelly, P. (2000). Inference of population structure using multilocus genotype data. Genetics, 155(2), 945–959. 10835412 10.1093/genetics/155.2.945 PMC1461096

[ece34589-bib-0043] Ronikier, M. (2011). Biogeography of high‐mountain plants in the Carpathians: An emerging phylogeographical perspective. Taxon, 60(2), 373–389.

[ece34589-bib-0044] Schmitt, T. (2007). Molecular biogeography of Europe: Pleitocene cycles and postglacial trends. Review. Frontier in Zoology, 4, 11 Biomed Central. https://www.frontiersinzoology.com/content/4/1/11 10.1186/1742-9994-4-11 PMC1868914 17439649

[ece34589-bib-0045] Schönswetter, P. , Stehlik, I. , Holderegger, R. , & Tribsch, A. (2005). Molecular evidence for glacial refugia of mountain plants in the European Alps. Molecular Ecology, 14, 3547–3555. 10.1111/j.1365-294X.2005.02683.x 16156822

[ece34589-bib-0046] Shaw, J. , Lickey, E. B. , Beck, J. T. , Farmer, S. B. , Liu, W. , Miller, J. , & Siripun, K. C. (2005). The tortoise and the hare II: Relative utility of 21 noncoding chloroplast DNA sequences for phylogenetic analysis. American Journal of Botany, 92(1), 142–166. 21652394 10.3732/ajb.92.1.142

[ece34589-bib-0047] Silvestro, D. , & Michalak, I. (2012). RaxmlGUI: A graphical front‐end for RAxML. Organisms Diversity and Evolution, 12(4), 335–337. 10.1007/s13127-011-0056-0

[ece34589-bib-0048] Simmons, M. P. , & Ochoterena, H. (2000). Gaps as characters in sequence‐based phylogenetic analyses. Systematic Biology, 49(2), 369–381. 10.1093/sysbio/49.2.369 12118412

[ece34589-bib-0049] Skrede, I. , Eidesen, P. B. , Pińeiro Portela, R. , & Brochmann, C. (2006). Refugia, differentiation and postglacial migration in arcticalpine Eurasia, exemplified by the mountain avens (*Dryas octopetala *L.). Molecular Ecology, 15, 1827–1840. 10.1111/j.1365-294X.2006.02908.x 16689901

[ece34589-bib-0050] Taberlet, P. , Coissac, E. , Pompanon, F. , Gielly, L. , Miquel, C. , Valentini, A. , … Willerslev, E. (2007). Power and limitations of the chloroplast trnL (UAA) intron for plant DNA barcoding. Nucleic Acids Research, 35(3), e14 10.1093/nar/gkl938 17169982 PMC1807943

[ece34589-bib-0051] Taberlet, P. , Fumagalli, L. , & Wust‐Saucy, A. G. (1998). Comparative phylogeography and postglacial colonization routes in Europe. Molecular Ecology, 7, 453–464. 10.1046/j.1365-294x.1998.00289.x 9628000

[ece34589-bib-0052] Taberlet, P. , Gielly, L. , Pautou, G. , & Bouvet, J. (1991). Universal primers for amplification of 3 noncoding regions of chloroplast DNA. Plant Molecular Biology, 17, 1105–1109. 10.1007/bf00037152 1932684

[ece34589-bib-0053] Thomson, J. D. , Higgins, D. G. , & Gibson, T. J. (1994). CLUSTAL W: Improving the sensitivity of progressive multiple sequence alignment through sequence weighting, position‐specific gap penalties and weight matrix choice. Nucleic Acids Research, 22(22), 4673–4680. 10.1093/nar/22.22.4673 7984417 PMC308517

[ece34589-bib-0054] Tribsch, A. , & Schönswetter, P. (2003). Patterns of endemism and comparative phylogeography confirm palaeo‐environmental evidence for Pleistocene refugia in the Eastern Alps. Taxon, 52, 477–497. 10.2307/3647447

[ece34589-bib-0055] Yanbaev, Y. A. , Bairamgulov, N. R. , Redkina, N. N. , & Mullagulov, R. Y. (2007). Differentiation among populations of the *Rhodiola iremelica* Boriss. (Crassulaceae) in the Southern Urals. Russian Journal of Genetics, 43(11), 1314–1318. 10.1134/s1022795407110154 18186196

[ece34589-bib-0056] Zhang, J. Q. , Meng, S. Y. , Allen, G. A. , Wen, J. , & Rao, G. Y. (2014). Rapid radiation and dispersal out of the Qinghai‐Tibetan Plateau of an alpine plant lineage *Rhodiola* (Crassulaceae). Molecular Phylogenetics and Evolution, 77, 147–158. 10.1016/j.ympev.2014.04.013 24780751

